# A Low Cost Device for Monitoring the Urine Output of Critical Care Patients

**DOI:** 10.3390/s101210714

**Published:** 2010-12-02

**Authors:** Abraham Otero, Francisco Palacios, Teodor Akinfiev, Andrey Apalkov

**Affiliations:** 1 Department of Information and Communications Systems Engineering, University San Pablo CEU, Boadilla del Monte 28668 Madrid, Spain; 2 Critical Care Unit, University Hospital of Getafe, Getafe, Carretera Toledo KM 12.500, 28901 Madrid, Spain; E-Mail: palaciosfra@gva.es; 3 Center of Automation and Robotics, Technical University of Madrid, Spanish Council for Scientific Research (CAR UPM-CSIC), La Poveda, Arganda del Rey, 28500 Madrid, Spain; E-Mails: teodor@iai.csic.es (T.A.); andrey.apalkov@iai.csic.es (A.A.)

**Keywords:** biosensors, urine output, critical care, intelligent alarms, patient monitoring

## Abstract

In critical care units most of the patients’ physiological parameters are sensed by commercial monitoring devices. These devices can also supervise whether the values of the parameters lie within a pre-established range set by the clinician. The automation of the sensing and supervision tasks has discharged the healthcare staff of a considerable workload and avoids human errors, which are common in repetitive and monotonous tasks. Urine output is very likely the most relevant physiological parameter that has yet to be sensed or supervised automatically. This paper presents a low cost patent-pending device capable of sensing and supervising urine output. The device uses reed switches activated by a magnetic float in order to measure the amount of urine collected in two containers which are arranged in cascade. When either of the containers fills, it is emptied automatically using a siphon mechanism and urine begins to collect again. An electronic unit sends the state of the reed switches via Bluetooth to a PC that calculates the urine output from this information and supervises the achievement of therapeutic goals.

## 1. Introduction

Nowadays, nearly any physiological parameter of a patient admitted to a critical care unit is sensed automatically by sophisticated commercial monitoring devices. This provides clinicians with invaluable information for interpreting the patient’s state. In most cases, these devices can also supervise whether the values of the physiological parameters they sense remain within a pre-established range set by the clinician. This range represents the values considered as acceptable for each parameter. If a parameter does not fall within its acceptable range, audible warnings to alert the health care staff are generated [1,2].

These devices discharge the healthcare staff of a considerable workload, since they need not continuously supervise whether the physiological parameters of every patient lie within the acceptable range. They also avoid human errors, which are common in any repetitive and monotonous task such as the supervision of physiological parameters [3,4].

Arguably, the most relevant physiological parameter which is still measured and supervised manually by healthcare staff is urine output. Urine output is the best indicator of the state of the patient’s kidneys. If the kidneys are producing an adequate amount of urine it means that they are well perfused and oxygenated. Otherwise, it is a sign that the patient is suffering from some complication. Urine output is required for calculating the patient’s water balance, which is essential in the treatment of burn patients [5]. Finally, it is also used in multiple therapy protocols to check whether the patient reacts properly to treatment [6]. When urine output is too low the patient is said to have oliguria. If the patient does not produce urine at all, then he/she is said to have anuria. Sometimes, urine output can be too high; in these cases the patient is said to have polyuria [7].

To measure urine output in critical care units, a Foley catheter is introduced through the patient’s urethra until it reaches his/her bladder. The other end of the catheter is connected to a graduated container that collects the urine. Periodically the nursing staff manually records the reading of the container of every patient, and operates a valve which releases the urine into a larger container. Thus, the healthcare staff does not benefit from the advantages of having a digital recording of this physiological parameter, or of the continuous and automatic supervision of its values.

In critical care units of first world countries, measurements of every patient’s urine output are taken hourly, 24 times a day, 365 days a year. In the case of emerging countries, often only burn patients—for whom urine output monitoring is of paramount importance—have this parameter recorded every hour, while the remaining critical patients have it recorded every 2 or 3 hours. This disparity in criteria is due to the more reduced availability of healthcare staff in the critical care units of emerging countries.

It can even be argued that the recording interval currently employed in first world countries—once every hour—is also a compromise between avoiding risk states for the patient and relieving the nursing staff from an excessive burden. A system capable of automatically monitoring urine output would decrease the workload associated with this task and, at the same time, permit supervision to take place on a more continuous basis.

On the other hand, there are abundant studies in the medical literature showing that critical patients’ outcome improves when the number of nurses [8–10] and the number of clinicians [11,12] available in the units increases. One of the factors that most influences the staffing of critical care units is the financial resources of the health system of each country [13]. Hiring highly qualified personnel, such as critical care unit staff, is very expensive. However, patient outcomes could also be improved by reducing the healthcare staff workload related to simple and repetitive tasks. This will free up time that could be used on other tasks.

From this point of view, the processes related to sensing and supervising urine output are a potential gold mine for saving time. Currently, in developed countries every hour a nurse must visit each of the patient’s bed of the critical care unit to manually record urine output. The nurse must put on gloves since he/she is going to manipulate body fluids, walk to the patient’s bed, take the measure visually, write it down, open the valve that releases urine from the graduated container to the plastic bag, wait for the urine to drain, close the valve, check that the valve is properly sealed and check if the plastic bag needs to be emptied. This procedure takes at least 2 minutes. In a critical care unit with 15 patients, this means 30 minutes per hour; 12 hours a day. Typically, nurses work in shifts of six hours. Therefore, each day two complete nursing shifts are required only for tasks related to supervising urine output. If these tasks were automated by a low cost device, we may obtain an improvement in patient outcomes equivalent to the improvement that would be obtained when two nurses per day are added to the staffing of the unit.

This paper presents a patent-pending device [14] capable of sensing and supervising urine output. Section 2 reviews related work. The device is described in Section 3. It uses reed switches that are activated by a magnet that is attached to a float in order to measure the amount of urine collected by two containers. An electronic unit checks the state of the reed switches and sends it to a PC, which supervises the achievement of the therapeutic goals that have been established for urine output. Section 4 analyzes the accuracy of the device. Section 5 explains how the therapeutic goals established by the clinician are supervised. Section 6 discusses the results of this work. Finally, a series of conclusions are given.

## 2. Related Work

The problem of automating urine output supervision has only begun to be tackled recently. There are several factors which probably have contributed to this. On the one hand, automatic urine output measurement requires a device capable of sensing very small amounts of liquid flow (as low as 3–5 milliliters per hour). This precludes the use of some common industrial solutions to measure the height of a column of liquid such as ultrasound sensors [15] or commercial flowmeters. On the other hand, any component of the device that is in contact, or may be in contact, with the urine cannot be reused on other patients and must be replaced approximately every 4–7 days for hygienic reasons. Therefore, components that can enter in contact with the urine must be easy to dispose of, and should have a low price. This precludes the use of expensive high precision laser based solutions [16].

Contact with the urine also means that the component is in indirect contact with the patient’s bladder through a Foley catheter. Therefore, the components that come into contact with the urine have to be sterilized before use. Finally, urine usually is acidic, although sometimes it can be slightly basic—its pH varies between 4.5 and 8. It contains Uric acid (between 25 and 75 mg/L), Urea (between 15 and 34g/24h), Sodium (between 130 and 260 mEq/24h), Potassium (less than 90 mEq/24h), Chlorine (between 110 and 250 mEq/24h), and Copper (less than 30 mcg/24h), among other components [17]. Thus, it can be corrosive, especially for metals.

The first device proposed to automatically measure urine output was Urinfo 2000, developed by the Israeli company Medynamix [18]. Urinfo 2000 was designed to automate the hourly urine output measurement, but not to take more frequent measures. Its operation is based on counting the number of drops of urine produced by the patient, and from this count it estimates urine output. The average error of the device when used to take hourly measurements was 8% (*±*25 mL). Urinfo 2000 cannot supervise therapeutic goals and its readings are not transmitted to a central station/PC. Thus it requires the nursing staff to take the measures from the device’s display, which is placed next to the patient’s bedside. At present, Medynamix has been bought by Flowsense, a construction services company that is starting to market the device [19].

The authors of this paper proposed a device to automate the supervision of urine output in [20]. Several technical and legal problems precluded us from moving this device beyond the laboratory validation phase. Using the knowledge we gained building it, we developed a second device with the main objective of conducting a series of clinical studies based on a more continuous and accurate monitoring of urine output throughout the stay of patients in a critical care unit [21,22]. This device uses a high precision scale to measure the weight of a commercial urine meter. On the scale’s pan there is a support frame made up of Bosch profiles that isolates the scale from force transmission from the patient’s bed, and guarantees that the urine flows properly through the urine meter’s input tube. The maximum measurement error of this device is under 1.5%.

Currently this device is in use at a research unit associated with the University Hospital of Getafe, in Spain. In this research unit a series of experiments aimed at the study of sepsis in an animal model—pigs—are being conducted. Sepsis is a serious medical condition characterized by a systemic inflammatory state that has developed in response to an infection and affects the patient’s entire body. Patients affected by this pathology are treated in an intensive care unit, and often require dialysis to support the functioning of the kidneys [23]. In these experiments, sepsis is induced by the administration of Escherichia coli bacteria. Animals are anesthetized and mechanically ventilated via tracheostomy. Blood pressure, pulmonary arterial pressure, renal artery flow and central flow are monitored invasively during the experiment. Urine output is continuously monitored by our device [21].

The aim of these experiments is twofold. On one hand, we would like to gain a better understanding of the pathophysiological mechanisms underlying systemic and renal hemodynamics during sepsis—hence the interest in a continuous and accurate monitoring of urine output. On the other hand, we would like to define the optimum monitoring interval for urine output, and to determine the level of accuracy required for this task. The high accuracy and acquisition rate of the device used in the experiments make it ideal for carrying out clinical studies. However, this device was not intended to be used in the clinical routine. Its size and operation make it somewhat tedious to be used in a critical care unit. Conversely, using this device we have learned that its accuracy (1.5%) and measurement interval (up to 10 s) are superior to what is required for properly supervising urine output. The data we have gathered so far suggests that an accuracy of 5% and a monitoring interval between 5 and 10 minutes are enough. Therefore, these parameters can be relaxed in order to build a simpler, more compact and cheaper device. These are the goals we are trying to achieve with the device presented this paper.

## 3. Measuring the Amount of Liquid that Flows

The patent that we have applied for describes a general sensor and procedure for measuring the amount of fluid flowing through a tube [14]. This procedure is more general than the one implemented by the device we have built to measure urine output. The following subsection covers the general sensor and procedure, and the next one presents the prototype we have built for the application that concerns us.

### 3.1. General sensor and procedure

**Figure 1.**
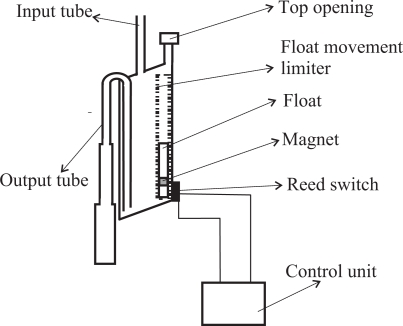
Diagram of the container that needs to be inserted in the tube through which the liquid is flowing.

Our goal is to measure the amount of fluid flowing through a tube. To this end, we insert a container somewhere in the tube in such a way that fluid enters the container through its top. The container is equipped with an output tube that empties its contents when it gets filled by taking advantage of the siphon principle (see Figure 1). One end of the output tube will be placed in the bottom wall of the container. Ideally the bottom wall will be inclined or conical and the tube will be in the lowest part. This favors a more complete evacuation of the container. Both ends of the output tube are always open. When the container fills, the liquid also goes up inside of the output tube until it reaches the elbow located in its top part. When it reaches the elbow, liquid begins to fall down the portion of the tube located on the exterior of the container. If the diameter of the output tube is chosen properly, all the liquid will flow down the output tube thanks to the principle of the siphon.

If the output tube is too wide, the siphon principle will not apply because when the water starts flowing, air could rise through the tube towards the container, stopping the evacuation. On the other hand, to drain the container as quickly as possible, it is desirable that the tube be as wide as possible. A compromise can be achieved using a variable diameter tube, so that its diameter grows (staggered or progressively) from the elbow of the tube to the end which is outside the container (see Figure 1). In this way, the principle of the siphon will work properly because the tube is narrow enough to prevent air flow towards the container in its upper part. But when the water begins to fall it finds a wider tube which provides less resistance, thereby increasing flow rate and decreasing the draining time.

The container must also be equipped with a top opening that is used to equalize the internal and external pressures. In the case of measuring urine output, this opening must be equipped with a filter to avoid the risk of bacterial contamination. The opening should be located at a height *H*_1_ above the elbow of the output tube. The liquid will not start flowing immediately when it reaches the elbow height; but it will continue to accumulate in the container until the height of the column of liquid which is above the elbow height overcomes the surface tension of the liquid against the walls of the output tube. The surface tension increases as the diameter of the output tube decreases. The height *H*_1_ and the *effective volume of the container V* *^S^* (the volume of water contained just before the drain starts) can be determined experimentally.

Ideally, the upper wall of the container will also be inclined or conical in shape. The top opening to equalize the internal and external pressures should be placed in the uppermost part of the wall (see Figure 1). This prevents bubbles from forming in the upper wall of the container, bubbles that would occupy volume and distort the measures.

**Figure 2.**
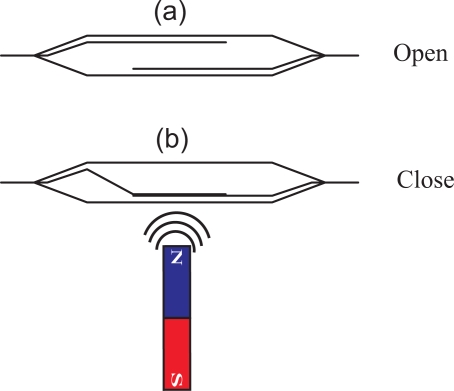
Operation of a reed switch. In the presence of a magnetic field, the switch is closed. In the absence of magnetic field, it is open.

On the outside wall of the container one or more reed switches are placed. A reed switch is an electrical switch which can be operated by a magnetic field. It contains two or more magnetizable, flexible, metal reeds hermetically sealed in a tubular glass envelope whose end portions are separated by a small gap. Under these conditions, the switch is open (see Figure 2(a)). A magnetic field properly applied will cause the reeds to bend, and the contacts to pull together, thus closing the switch (see Figure 2(b)).

A float within a structure designed to limit its movement so that it can only move vertically is located inside of the container, near the container wall where the reed switches are placed (see Figure 1). The float has a magnet attached which interacts with the reed switches closing them when the magnet is approximately at the same height as each of the reed switches. The state of the switches is continuously checked by an electronic unit.

Let us suppose that there are *N* reed switches on the outside wall of the container. The procedure for measuring the volume of liquid flowing into the container is as follows. At least one reed switch should be located in a position such that when the container is empty, the magnet located on the float closes that reed switch. As the liquid begins to flow into the container, the float, and therefore the magnet, begins to rise. At some point, the magnet will stop interacting with the first reed switch and, therefore, it opens. At that point, a volume *V*_1_ of liquid has flowed into the container. When enough liquid has accumulated in the container, the magnet will rise up to the point where it closes the second reed switch. At this point an additional volume *V*_2_ of liquid has flowed into the container, being the total amount of liquid accumulated *V*_1_ +*V*_2_. When the magnet moves higher, the second reed switch opens again and an additional volume *V*_3_ of liquid has flowed –being the total volume of liquid *V*_1_ + *V*_2_ + *V*_3_.

In general, when the reed switch *n* is closed, 1 < *n* ≤ *N*, we will add the volume *V*_2_*_n−_*_2_ to the the volume of liquid that has flowed, and when the reed switch *n* is opened again, we will add the volume *V*_2_*_n−_*_1_. When the container is emptied, the float with the magnet will go back to the bottom of the container, and therefore it will close the first reed switch. At this point, the effective volume of the container—*V* *^C^*—has flowed, and the measurement procedure is resumed. The volumes *V*_2_*_n−_*_2_ and *V*_2_*_n−_*_1_ for each reed switch, and the effective volume *V* *^C^* can be determined experimentally.

This measurement procedure has a flaw: the volume of liquid that flows into the container from the time the container begins to empty through the siphon mechanism, until it is completely empty, will not be measured. Depending on the specific application and characteristics of the container—mostly its draining time—this may or may not be tolerable. This is the reason why it is important to design carefully the output tube of the small container, choosing an appropriate tube’s diameter that grows staggered or progressively from the elbow of the tube to the end which is outside the container. In this way we can minimize the draining time.

**Figure 3.**
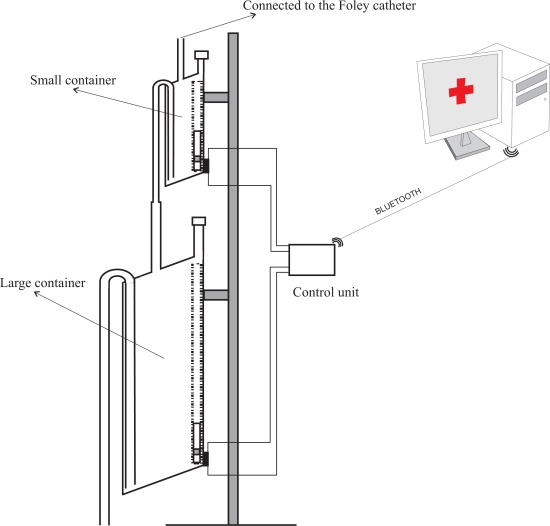
Diagram of our device. Each of the containers follows the design shown in Figure 1.

However, this problem can be solved using two different size containers, each of them working according the same principles presented here. Both containers would be connected in cascade with the output of the smaller container connected to the input of the larger one (see Figure 3). Thus, although the volume of liquid that flows during the discharge of the first container is not measured by the small container, it will be measured by the larger one.

The chaining of the two containers causes a new problem. It can happen that when the small container releases its content, the larger one is nearly full. In the worst case scenario, the first drop that falls from the small container would trigger the draining of the large container. Thus, almost all the content of the small container would not be measured in the large one. The maximum measurement error this may cause is (*V* *^S^/V* *^L^*)%, being *V* *^S^* and *V* *^L^* the effective volumes of the small and large containers, respectively. On average, the large container will start to drain when the small container is emptied halfway. Therefore, the average error caused by this effect will be ((*V* *^S^/*2)*/V* *^L^*)%. As long as *V* *^L^* ≫ *V* *^S^*, this error will be small. Therefore, for each concrete application the volumes of both containers can be adjusted so that this error is negligible.

### 3.2. Our prototype

**Figure 4.**
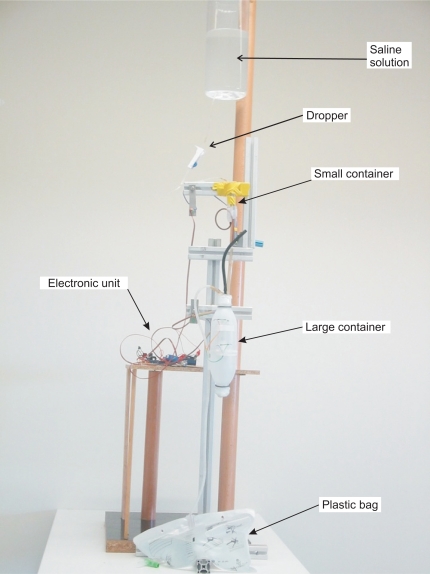
Picture of the prototype device with the saline solution and eye dropper used in its validation.

In our prototype the fluid urine flows into the container through a flexible plastic tube which is connected to the Foley catheter—the catheter which is introduced through the patient’s urethra up to the bladder. Monitoring the amount of urine produced by a patient requires an early warning about deviations from therapeutic goals. Thus, a small volume container must be used—approximately 5 mL in the prototype we have built. A container of such a small size is likely to evacuate its contents many times throughout the day, which could lead to considerable measurement errors due to the inability to measure the liquid that flows during the container discharge. Therefore, we have opted for using two containers in the prototype. The first one was built to have a volume of approximately 5 mL, and the second a volume of approximately 375 mL (see Figure 4). These volumes ensure that small measurement errors will occur if the second container evacuates before the first container is emptied completely, given that 375 ≫ 5.

The first container is equipped with a reed switch that is activated when the float is on its lowest position. The staggered diameter of its output tube increases in three steps. The output tube is connected to the input tube of the larger container, which is equipped with another reed switch. Given the larger size of the second container, it would be easy to locate several reed switches on its wall to detect intermediate filling points. We built a first prototype that employed more than one reed switch in the second container. However, during its validation we found that a single reed switch results in a device with more than enough accuracy for our application. Thus we decided to simplify the device minimizing the total number of reed switches.

In the prototype, the output tube of the large container is connected to a 2.5 liter plastic bag that collects the liquid once it has been measured (see Figure 4). An electronic unit continuously checks the status of the reed switches and reports any change via Bluetooth to a Java application that runs on a PC. From these changes, and from the effective volumes of the containers, the application calculates the volume of urine that has flowed and displays a chart with this information.

## 4. Calibration and Accuracy of the Device

The effective volume of the containers—the volume of liquid that triggers the emptying through the siphon mechanism—is slightly higher than the volume corresponding to the height of the top of the siphon mechanism because the liquid does not begin to flow until the pressure overcomes the surface tension force of the liquid against the walls of the output tube. The effective volumes of the small container (*V* *^S^*) and of the large container (*V* *^L^*) were determined experimentally, each of them separately. A saline solution with properties similar to those of urine and an dropper were used to simulate the flow of urine (see Figure 4). The containers were placed so that they would release their content on a bowl placed on the plate of a high-precision industrial scale—a PGW 4502e, built by Adam Equipment Inc. This scale has an accuracy guaranteed by the manufacturer of 0.01 g. Given that the density of the saline solution was known, we can determine the volume of liquid that the container releases into the bowl from the weight.

The PGW 4502e scale is equipped with a serial port that permits querying for readings. We built a program that periodically takes measurements from the scale. The program together with the dropper allow us to automate the process of carrying out multiple measures of the volume of liquid released by the containers. Using this set up we took 500 measurements of the volume of liquid released by the small container, and 100 measures of the volume of liquid released by the large container. From these measures we calculated the effective volumes of the containers: 5.82 *±* 0.32 mL and 376.52 *±* 1.14 mL (mean *±* standard deviation).

**Figure 5.**
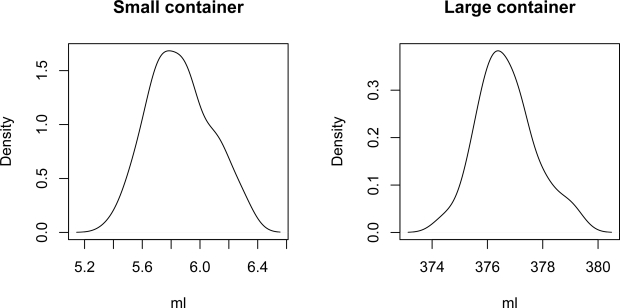
Kernel density estimates of the effective volume distribution of the small and large containers. Given these plots, it seems reasonable to assume that both distributions are normal.

Figure 5 shows kernel density estimates [24] of the effective volume distributions of both containers obtained from the calibration measurements. It seems reasonable to assume that both distributions are normal. Thus, given that the effective volume of the small container is 5.82 *±* 0.32 mL, in 95% of cases the amount of urine that is released when the container is emptied will be in the range 5.82 *±* 0.64 mL (mean *±* 2· standard deviation). Therefore, 95% of the measures derived from the small container will have an error of less than 10.9%. The purpose of this small container is to provide an early warning if the patient’s urine output is not within the therapeutic goals: depending on the patient’s weight and state this container should be filled between 5 and 12 minutes, if the patient is within the therapeutic goals.

Assuming that the small container effective volume distribution is normal, after taking *m* measures the expected value for the amount of liquid that has flowed is *m* · 5.82, and the standard deviation of the aggregate result of these measures is $0.32/\sqrt{m}$. For example, for *m* = 10, the volume of liquid that would have flowed would be 58.7 mL *±* 0.10 mL. Therefore, in 95% of cases the amount of urine that has been produced would be in the range 58.7 mL *±* 0.20 mL, with a maximum measurement error 0.34%.

According to this estimate, although the error in each of the small container measures can be high—up to 10.9%, this error decreases quickly when several measures are taken. However this estimate does not take into account that the liquid that has flown during the emptying of the container cannot be measured. The magnitude of this second source of error has been estimated experimentally. To this end, we simulated different rates of urine production using a dropper and we compared the difference between the liquid flow estimated from the number of times the container is emptied, and the measures of the high precision scale PGW 4502e.

Six different rates of urine production were simulated: approximately 100, 250, 500, 1,000, 2,000, and 4,000 mL/day. Although the precise interpretation of these values depends on the patient’s condition and weight, usually 100 and 250 mL of urine per day correspond to severe oliguria. 500 mL/day corresponds to moderate oliguria. 1,000 and 2,000 mL/day are normal—healthy—amounts. 4,000 mL/day corresponds to polyuria. For each of these urine production rates three different experiments, each with a duration of 24 hours, were carried out. After 24 hours, we obtained the measurement error as the difference between the estimated urine output and the one calculated from the scale’s measures. The results are shown in the second and third columns of Table 1. As expected, when doubling the urine production rate, the error increases by a factor of approximately 4. Doubling the production rate doubles both the amount of fluid that is not measured during the draining of the container and the number of times the container needs to be drained.

For small amounts of urine the error is acceptable. Extrapolating from the results of the table, at a urine production rate of approximately 1,700 mL/day, the error is no longer acceptable; *i.e.*, it exceeds 5%, which according to the data we have gathered in experiments with animal models [21] is the maximum permissible error for urine output measurement.

However, the second container allows us to correct the measurements each time it is drained; *i.e.*, all measures of Table 1 except the first two could have been corrected using information from the larger container. In 95% of cases, the amount of urine released by this container will be within the range 376.52 *±* 2.28 mL (mean *±* 2·standard deviation). On the other hand, on average half of the effective volume of the small container will not be registered. Thus, the average error in the measures will be 5.82/2 + 2.28 = 5.19; 1.38% of its volume. The maximum error, which would correspond to the case in which the first drop from the small container starts the draining of the large container, will be 5.82 + 2.28 = 5.19; 2.15% of its volume.

Therefore, once 376.52 mL of urine have been produced, it would be possible to reduce the average measurement error down to 1.38%. The fourth column of Table 1 shows the maximum error in the measurements of the small container for each urine production rate just before the first correction with information from the large container was applied. The fifth column shows this error as a percentage of the total urine production.

The total urine output (*UO*) produced at each instant is given by:

(1) $$UO=n\cdot V^{L}+m\cdot V^{S}$$

where *n* is the total number of times the large container has been evacuated, and *m* is the number of times the small container has been evacuated since the last time the large container was drained. The measurement error when *n* = 0 or when *m* = 0 has already been discussed. The error when *n* > 0 and *m* > 0 is given by:

(2) $$\frac{2\cdot \left(\frac{\sigma_{V^{L}}}{\sqrt{n}}+\frac{\sigma_{V^{S}}}{\sqrt{m}}\right)+n\cdot \frac{V^{S}}{2}}{n\cdot V^{L}+m\cdot V^{S}}$$

the term $\frac{\sigma_{V^{L}}}{\sqrt{n}}+\frac{\sigma_{V^{S}}}{\sqrt{m}}$ is the standard deviation of the aggregate result of the measures of both containers. The factor 2· is used to obtain a confidence interval of 95% in the error of the aggregate measures. The term $n\cdot \frac{V^{S}}{2}$ accounts for the fact that, on average, each time the large container is emptied half of the volume of the small container is not measured. The denominator of the expression is the total urine output.

Equation 2 is a monotonically decreasing function both in *n* and *m*. Given that Equation 2 only applies when *n* > 0 and *m* > 0 *,* its maximum occurs when *n* = *m* = 1. In the case of our prototype, the maximum error is 1.5%. Therefore, the maximum error in the measurements of the device still occurs when a single measure from the small container is available.

## 5. Therapeutic Goals Supervision

An electronic unit sends the state of the reed switches which monitor the containers’ evacuations to a Java application installed on a PC via Bluetooth. From these states and from the values of *V* *^S^* and *V* *^L^*, the amount of liquid flow can be calculated using Equation 1. The Java application allows the healthcare staff to inspect a graph showing urine output, and to set its therapeutic goals. These therapeutic goals are represented with the aid of the Fuzzy Set Theory, a tool which has proved its value for handling and representing medical knowledge [25].

We shall introduce some basic concepts of the Fuzzy Set Theory. Given a discourse universe ℝ we define a *fuzzy value C* by means of a possibility distribution *π^C^* defined over ℝ [26]. Given a precise value *x ∈* ℝ*, π^C^*(*x*) *∈* [0*,* 1] represents the possibility of *C* being precisely *x*. A *fuzzy number* [27] is a normal (*∃x ∈* ℝ*, π^C^*(*x*) = 1) and convex (*∀ x, x′, x″ ∈* ℝ, *x′ ∈* [*x, x^″^*], *π^C^*(*x′*) *≥ min*{*π^C^*(*x*)*, π^C^*(*x″*)}) fuzzy value. Normality and convexity properties are satisfied by representing *π^C^*, for example, by means of a trapezoidal representation. In this way, *C* = (*α, β, γ, δ*), *α ≤ β ≤ γ ≤ δ*, where [*β, γ*] represents the core, *core*(*C*) = {*x ∈* ℝ*| π^C^*(*x*) = 1}, and ]*α, β*[ represents the support, *supp*(*C*) = {*x ∈* ℝ*|π^C^*(*x*) *>* 0}.

A fuzzy number *C* can be obtained from a flexible constraint given by a possibility distribution *π^C^*, which defines a mapping from ℝ to the real interval [0*,* 1]. A fuzzy constraint can be induced by an item of information such as *“x has a high value”*, where *“high value”* will be represented by *π^C^*^=^*^high^*. Given a precise number *x ∈* ℝ, *π^C^*^=^*^high^*(*x*) *∈* [0*,* 1] represents the possibility of *C* being precisely *x; i.e.*, the degree with which *x* fulfills the constraint induced by *“high value”*.

**Figure 6.**
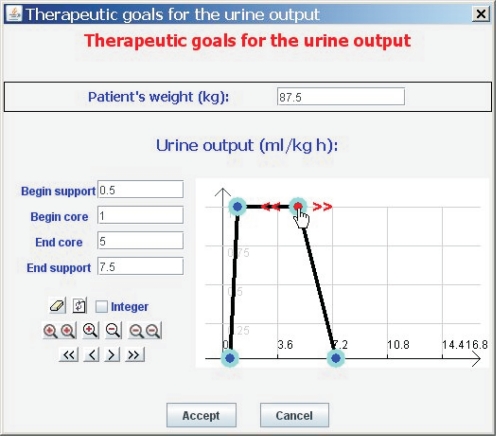
Window that allows healthcare staff to set the therapeutic goals for urine output.

**Figure 7.**
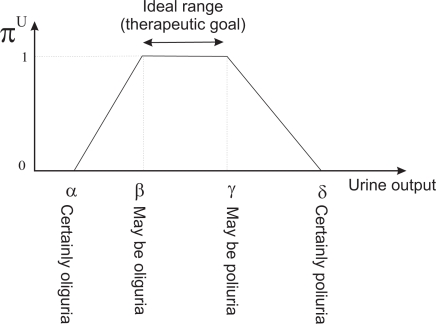
The therapeutic goals represented by the trapezoidal possibility distribution.

Clinicians are accustomed to expressing the therapeutic goals for urine output in milliliters of urine produced per kilogram of patient body mass per hour—*ml/kg* · *h*. Our tool allows them to indicate the weight of the patient (*P*) and the therapeutic goals represented by the trapezoidal possibility distribution *π^U^* (see Figure 6). *π^U^* can be interpreted as a computational projection of the piece of clinical knowledge “adequate UO”. The minimum and maximum values acceptable for urine output are the beginning and the ending of the support of the distribution, respectively. If the patient produces less urine than the amount corresponding with the beginning of the support, the patient is clearly in oliguria. If he/she produces more urine than the amount corresponding with the ending of the support, the patient is clearly in polyuria. The beginning and ending of the core are the limits of the interval within which ideal values of urine output lie (see Figure 7).

If *u_i_* is the urine output in *ml/kg* · *h*, the degree to which the therapeutic goals established by the clinician are being met is *π^U^*(*u_i_*). If *π^U^*(*u_i_*) = 1 then the urine output is within the range of ideal values. If *π^U^*(*u_i_*) = 0 then either the urine output is less than the minimum acceptable value (the patient has oliguria or anuria), or greater than the maximum acceptable (the patient has polyuria). The closer *π^U^*(*u_i_*) is to 1, the closer the amount of urine produced by the patient is to the ideal value, and the closer *π^U^*(*u_i_*) is to 0, the closer the patient is to oliguria or polyuria.

In the tool *π^U^*(*u_i_*) is represented by a color code used when drawing the graph of urine output. Red represents the null compatibility (the patient is clearly in oliguria or in polyuria), followed by orange, yellow, green and black, which represents the total compatibility (the urine output lies within the range of ideal values). Therefore, the urine output graph provides instant visual feedback on the patient’s state [21].

The tool generates an audible warning when the small container is not filled within the maximum time allowed by *π^U^*; *i.e.*, when the patient clearly has oliguria. The time at which the alarm is triggered is given by:

(3) $$t_{\text{alarm}\;\text{oliguria}}=\alpha \cdot P/\left(V^{S}+2\cdot \sigma_{V^{S}}\right)$$

where *α* is the beginning of the support of *π^U^*, *P* is the patient’s weight, *V* *^S^* is the effective volume of the small container, and *σ_V^S^_* is the standard deviation of the effective volume of the small container. The correction *V* *^S^* + 2 · *σ_V ^S^_* is applied to avoid false positives caused when the small container does not release its contents when urine output reaches its average effective volume, but some more urine accumulates before releasing.

Similarly, the tool also produces an audible warning when the small container fills faster than the minimum time allowed by *π^U^*; *i.e.*, when the patient clearly has polyuria. This alarm is triggered when the small container fills before the time given by:

(4) $$t_{\text{alarm}\;\text{polyuria}}=\delta \cdot P/\left(V^{S}-2\cdot ,\;\sigma_{V^{S}}\right)$$

where *δ* is the end of the support of *π^U^*. The correction *V* *^S^* *−* 2 · *σ_V ^S^_* is applied to avoid false positives caused when the small container releases its contents before reaching its effective volume.

## 6. Discussion

The measurement accuracy of our device depends on the urine production rate of the patient. As Table 1 shows, the smaller the rate is, the more accurate the measurements are. This behavior suits very well the nature of the problem we are addressing: for patients that produce normal amounts of urine, or that have polyuria, it is not required to have a high degree of accuracy in the measurement of the urine output. The more urine is produced, the less important it is to have an accurate measure; only when the patient is producing small amounts of urine is it important to measure accurately [21].

The maximum uncertainty in the measurements occurs when a single measure of the small container is available. Given that its effective volume is 5.82 *±* 0.32 mL, with a confidence interval of 95% the measurement error will be under 10.9%. After taking *m* measures from the small container the expected value for the amount of liquid that has flowed is *m ·* 5.82, and the standard deviation of the aggregate result of these measures is $0.32/\sqrt{m}$. Therefore, for *m* = 2, the maximum error with a confidence interval of 95% will be 3.9%; already under 5%. As Equation 2 shows, when the number of measurements from both containers increases, the measurement error decreases. Therefore, only when a single measure from the small container is available the measurement error exceeds 5%. If the patient is producing urine within the therapeutic goals, the time during which the error exceeds 5% should be below 20 minutes. If the patient has oliguria, although the container may not fill within this time, the oliguria alarm will be triggered before 15 minutes, providing feedback about the patient’s condition.

These estimates do not take into account the error caused by the inability to measure urine production during the draining of the small container. This error grows with the number of measures taken from the small container. However, as Table 1 shows, it is negligible if the patient’s urine output is low, and it can be corrected each time the large container is emptied.

One might think that the measurement error caused by the inability to measure the urine produced while the small container is drained could be corrected on a per small container evacuation basis rather than per large container evacuation. When the large container is evacuated once, we could estimate how much urine has flowed during these intervals, and use this estimate to correct future measures of the small container. However, the urine production rate of the patient (and therefore the amount of urine that flows during the draining intervals) can vary rapidly, hence the interest in a continuous monitoring of urine output. Furthermore, in patient monitoring one should always lean towards the side of caution. A system which considers that the production of urine during the small container draining is the worst case scenario for the patient’s health (zero) it is preferable to a system that may be more accurate, but that under certain conditions may overestimate urine output, and therefore could lead clinicians to think the patient is in a better state than he/she really is. Hence we made the decision not to apply any correction.

The measurement error of the device is very low when compared to errors committed by the nursing staff when they take measures visually. For example, in the commercial urine meter Unometer 500—the one used in the ICU of the hospital we collaborate with—the separations between the coarser measurement divisions in the 500 mL graduated container where urine is collected correspond with 20 mL. Therefore, the minimum measurement error is 4%. However, this is a highly optimistic estimation of the error. It assumes that the visual measurements are taken in ideal conditions; *i.e.*, the graduated container is not tilted in any direction and the nurse’s eyes are in the same horizontal plane as the graduated container. In practice, the average visual measurements error has been reported to be as high as 26% [18].

Our device provides feedback on the status of the patient’s kidneys more frequently than is currently available in critical care units –hourly. For example, for a patient of 80 kilos, which should produce at the very least 40 milliliters of urine per hour, our device could warn of a deviation from the therapeutic goals in approximately 9 minutes. This allows the clinician to react more promptly to complications in the state of the patient. Therefore, the device has the potential of improving patient outcomes.

The complexity of our device is not greater than the complexity of commercial urine meters, which often require a small container embedded within a large container, valves that communicate both containers with each other and with a plastic bag, mechanisms to prevent the containers from overflowing, filtered openings to equalize pressures, etc. The materials required to build our prototype are also similar to the ones required for commercial urine meters, with the exception of the additional need of two reed switches, two floats and two magnets. The rest of the pieces that are part of our solution do not have to be discarded because they are not in contact with the patient’s urine, nor do they suffer significant degradation caused by its operation. Thus, their cost can be amortized over a long period of time and their impact on the overall cost of the solution is negligible. Therefore, the price of mass production of our device should be only slightly higher than the commercial urine meters price.

An automatic supervision of the patient’s urine output can also improve patient outcomes indirectly by reducing the healthcare staff workload. As we argued in the introduction of the article, in a critical care unit of a developed country with 15 patients, approximately 12 hours per day are necessary for tasks related to supervising urine output. Typically, nurses work in shifts of six hours. Therefore, each day two complete nursing shifts are required for these tasks. Automating the supervision of urine could produce an improvement in patient outcomes similar to the improvement obtained if two nurses per day were added to the staffing of the unit [8,9].

In the contexts where the additional cost of the device could be a barrier to adoption, the institutions that provide healthcare services may opt for a staff reduction to offset the higher costs of the device. In this way, a more precise and continuous monitoring of urine output would be obtained without an increase in costs.

## 7. Conclusions

We have presented a device capable of automatically sensing and supervising the urine output of critical care patients. The device comprises two containers of different volumes, a small one that receives the urine coming from the patient’s bladder, and a greater volume container in which the first container releases its content when it gets full. Both containers release their content automatically when they are filled using a siphon mechanism. The containers are equipped with reed switches that are activated by a magnet that is attached to a float located inside the containers. These reed switches allow us to identify the instants at which the containers get filled with urine. An electronic unit sends via Bluetooth the information provided by the reed switches to a PC which calculates the urine output from the switches’ state, and supervises the achievement of the therapeutic goals established by the clinician.

The error in the measures of the device depends on the patient’s urine production. For most common scenarios, it is below 2%. This is significantly lower than the error committed by the nursing staff when they take measures visually. In addition, the device provides feedback on the status of the patient’s kidneys every 5 to 10 minutes (depending on the patient’s weight), while currently that information is available hourly. This early warning of deviations from the therapeutic goals could translate into improved patient outcomes.

The cost of mass production of the device is slightly higher than the cost of the commercial urine meters currently in use. However, the device frees a considerable amount of time for the healthcare staff. The time saving can yield cost savings to the institutions that provide health services. Conversely, the saved time can be repurposed for more relevant tasks, and thus it can generate an improvement in patient outcomes.

The overall result is a low cost device that permits both a more precise and more continuous monitoring of urine output than is currently possible in critical care units, and that therefore has the potential of improving patient outcomes. The next step is to take the device to the clinical routine. This will require a commercial partner capable of producing the device in the appropriate sterile conditions and distributing it to the hospitals.

## Figures and Tables

**Table 1.** Average errors for each of the urine production rates with and without correction. In cases marked with an * the large container correction cannot be applied.

Flow rate (ml/day)	24h average error	% error	Max. error before correction	%Max. error
100	0.23	0.23%	0.23*	0.23%*
250	1.57	0.63%	1.57*	0.63%*
500	6.89	1.32%	5.19	1.04%
1,000	29.4	2.94%	11.1	1.11%
2,000	122	6.10%	23.0	1.15%
4,000	513	12.8%	48.3	1.21%

## References

[1] Otero A, Félix P, Barro S, Palacios F (2009). Addressing the flaws of current critical alarms: A fuzzy constraint satisfaction approach. Artif Intell Med.

[2] Hande A, Polk T, Walker W, Bhatia D (2006). Self-powered wireless sensor networks for remote patient monitoring in hospitals. Sensors.

[3] Jungk A, Thull B, Rau G (2002). intelligent alarms for anaesthesia monitoring based on fuzzy logic approach. Fuzzy Logic in Medicine.

[4] Mora F, Passariello G, Carrault G, Pichon JL (1993). Intelligent patient monitoring and management systems: A review. IEEE Eng Med Biol.

[5] Mitra B, Fitzgerald M, Cameron P, Cleland H (2006). Fluid resuscitation in major burns. ANZ J Surg.

[6] Rivers E, Nguyen B, Havstad S, Ressler J, Muzzin A, Knoblich B, Peterson E, Tomlanovich M (2001). The Early Goal-Directed Therapy Collaborative Group, Early goal-directed therapy in the treatment of severe sepsis and septic shock. New Engl J Med.

[7] Williams G, Pickup JC (2004). Handbook of Diabetes.

[8] Kovner C, Gergen P (1998). Nurse staffing levels and adverse events following surgery in US hospitals. J Nurs Scholarship.

[9] Amaravadi R, Dimick J, Pronovost P, Lipsett P (2000). ICU nurse-to-patient ratio is associated with complications and resource use after esophagectomy. Intens Care Med.

[10] Knauf R, Lichtig L, Risen-McCoy R, Singer A, Wozniak L (1997). Implementing Nursings Report Card: A Study of RN Staffing, Length of Stay and Patient Outcomes.

[11] Pronovost P, Angus D, Dorman T, Robinson K, Dremsizov T, Young T (2002). Physician staffing patterns and clinical outcomes in critically ill patients: A systematic review. Jama.

[12] Dimick J, Pronovost P, Heitmiller R, Lipsett P (2001). Intensive care unit physician staffing is associated with decreased length of stay, hospital cost, and complications after esophageal resection. Crit Care Med.

[13] Pronovost P, Needham D, Waters H, Birkmeyer C, Calinawan J, Birkmeyer J, Dorman T (2004). Intensive care unit physician staffing: Financial modeling of the Leapfrog standard*. Crit Care Med.

[14] Akinfiev T, Apalkov A, Otero A, Palacios F Device for measuring the amount of liquid that flows and procedure for its measurement.

[15] Johnson SJ (1978). Liquid Level Measurement Device.

[16] Ishida S (1990). Liquid level indicator using laser beam.

[17] Brunzel NA (1994). Fundamentals of Urine and Body Fluid Analysis.

[18] Hersch M, Einav S, Izbicki G (2009). Accuracy and ease of use of a novel electronic urine output monitoring device compared with standard manual urinometer in the intensive care unit. J Crit Care.

[19] Flowsensemedical Flowsensemedical website. http://www.flowsensemedical.com.

[20] Otero A, Panigrahi B, Palacios F, Akinfiev T, Fernández R, Naik G (2009). A prototype device to measure and supervise urine output of critical patients. Recent Advances in Biomedical Engineering.

[21] Otero A, Akinfiev T, Fernández R, Palacios F (2010). A device for automatically measuring and supervising the critical care patientS urine output. Sensors.

[22] Otero A, Akinfiev T, Fernández R, Palacios F (2009). A device for automatic measurement of critical care patient’s urine output. 6th IEEE International Symposium on Intelligent Signal Processing.

[23] Klenzak J, Himmelfarb J (2005). Sepsis and the kidney. Crit Care Med.

[24] Sheather S, Jones M (1991). A reliable data-based bandwidth selection method for kernel density estimation. J Roy Stat Soc B Stat Meth.

[25] Barro S, Marín R, Palacios F, Ruíz R (2001). Fuzzy logic in a patient supervision systems. Artif Intell Med.

[26] Zadeh L (1975). The concept of a linguistic variable and its application to approximate reasoning. Inform Sci.

[27] Kaufmann A, Gupta M (1984). Introduction to Fuzzy Arithmetic.

